# Integrative Analysis of the Physical Transport Network into Australia

**DOI:** 10.1371/journal.pone.0148831

**Published:** 2016-02-16

**Authors:** Robert C. Cope, Joshua V. Ross, Talia A. Wittmann, Thomas A. A. Prowse, Phillip Cassey

**Affiliations:** 1 School of Mathematical Sciences, The University of Adelaide, SA 5005, Australia; 2 School of Biological Sciences, Benham Laboratories, The University of Adelaide, SA 5005, Australia; CSIRO, AUSTRALIA

## Abstract

Effective biosecurity is necessary to protect nations and their citizens from a variety of threats, including emerging infectious diseases, agricultural or environmental pests and pathogens, and illegal wildlife trade. The physical pathways by which these threats are transported internationally, predominantly shipping and air traffic, have undergone significant growth and changes in spatial distributions in recent decades. An understanding of the specific pathways and donor-traffic hotspots created by this integrated physical transport network is vital for the development of effective biosecurity strategies into the future. In this study, we analysed the physical transport network into Australia over the period 1999–2012. Seaborne and air traffic were weighted to calculate a “weighted cumulative impact” score for each source region worldwide, each year. High risk source regions, and those source regions that underwent substantial changes in risk over the study period, were determined. An overall risk ranking was calculated by integrating across all possible weighting combinations. The source regions having greatest overall physical connectedness with Australia were Singapore, which is a global transport hub, and the North Island of New Zealand, a close regional trading partner with Australia. Both those regions with large amounts of traffic across multiple vectors (e.g., Hong Kong), and those with high levels of traffic of only one type (e.g., Bali, Indonesia with respect to passenger flights), were represented among high risk source regions. These data provide a baseline model for the transport of individuals and commodities against which the effectiveness of biosecurity controls may be assessed, and are a valuable tool in the development of future biosecurity policy.

## Introduction

The international transport of people and goods poses significant border security risks: transmission of infectious diseases [1, 2], individuals and commodities associated with transnational crime or terrorism [3], and threats to food security (agricultural pests or pathogens) or the environment [2, 4]. Trade, and associated airborne and seaborne traffic occurs on a global scale, and has increased in volume over recent decades. To effectively assign border security effort and manage risks, it is necessary to quantify physical connectedness between regions on a global scale, to identify transport-risk hotspots and variations over time. As invasion biologists, we approach border security and physical connectedness with the primary motivation of enhancing biosecurity protection, and, as such, much of this manuscript will come from this perspective.

Biological invasions present significant environmental and economic costs when invasive species become established outside their native geographic ranges [5, 6]. Global growth in transnational air travel and seaborne trade has played a significant role in increasing the rates of biological invasions [7], through aiding movement across natural geographic barriers at historically unprecedented rates [8, 9]. Human-mediated physical transport networks facilitate the introduction of alien species through a variety of pathways and means [10], which we group into three key categories: (i) intentional transport including trade and illicit smuggling, (ii) unintentional stowaways, and (iii) accidental transport alongside a commodity. Intentional transport (including illicit smuggling) of species and their subsequent release or escape contributes significantly to alien species introduction, e.g., the global pet trade in slider turtles has resulted in introductions in at least 73 countries worldwide [11]. Unintentional transport of stowaways has resulted in the introduction of over 500 exotic terrestrial plant and animal species globally [7], and rising volumes of ballast discharge associated with shipping traffic has been identified as the primary driver of marine invasions [12]. The third major pathway is accidental introduction alongside a commodity, e.g., pathogens and insect pests associated with host plants [7, 13]. The importation of timber products represents an extreme threat to the biosecurity of all forested countries, with historical quarantine data identifying large numbers of insect pests being detected in the transportation and trade of wood products [14, 15]. International trade in ornamental plants has accounted for the majority of insect invasions in Europe [16]. Increased pest interception rates have also been related to incoming air traffic volumes into the United States [17].

The establishment phase of the invasion framework (*sensu* Blackburn [18]) has been subject to the most research attention [19]. Many examples exist linking invasion events to particular pathways, however few studies have considered physical transport connectedness between regions over multiple pathways [9]. Targeted analyses of sea or air traffic have been performed independently (e.g., [20–23]), but very few studies have considered both air and seaborne traffic concurrently, and these have been primarily linked to disease vector dispersal (e.g., [24, 25]). Variations in traffic volumes across transport pathways makes combined traffic analysis complex, but determining common features is necessary for effective comparison between pathways [9]. Worldwide air passenger traffic has grown by nearly 9% per year since 1960, greater than twice the rate at which global average GDP has grown. Similar growth has also occurred in airfreight traffic [26]. Approximately 90% of total world trade, by volume, is carried by sea [27]. In 2012, 9.2 billion tonnes of goods were loaded in ports worldwide, compared to 8.8 billion tonnes in 2011, again exceeding the rate of global average GDP growth [28]. Given these increasing trends in sea and air traffic volumes, there is a growing need to determine specific pathways and donor-risk hotspots based on a combination of different forms of transport, providing vital information for the development of effective biosecurity strategies into the future.

Here, we use Australia as a case study for the analysis of the physical transport network, with respect to biosecurity surveillance and planning. Border biosecurity and biosurveillance schemes in Australia are often considered among the most stringent in the world [29], and an example of international best practice [30]. Australia is the world’s largest continental island and is uniquely isolated from land-based biosecurity incursion threats and biological invasions. Yet, even among widespread conspicuous taxa (e.g., alien vertebrates) new post-border incursions have continued to increase in the recent decade [31]. With air and seaborne traffic into Australia constantly increasing, invasion risk is likely to concomitantly increase, and biosecurity policy, directed towards reducing this risk, is of primary concern [30]. International passenger traffic into Australia increased by 71.2% between 2004 and 2014 [32]. Shipping traffic into Australia has increased significantly between 1999 and 2012, with the annual number of vessel arrivals increasing by more than 60%, and the average size of vessels also increasing [20]. Our findings will directly advance the understanding of the transport stage in the invasion pathway, and inform Government quarantine planning and prevention strategies to mitigate future alien species impacts through implementation of a risk-based approach to biosecurity management [33].

Effective monitoring and risk assessments are key components of biosecurity policy [30]. The individual importance of different transport pathways is likely to vary temporally, spatially, and by context (i.e., across taxa in an invasion context, between different pathogens in an infectious disease context, etc.), and thus it is important to have a flexible framework for quantifying physical connectedness between regions and identifying transport risk hotspots. In this study we have developed a framework, and associated online tool, which allows managers and policy makers to compare risk profiles and identify transport-source hotspots through prioritising shipping traffic and air traffic in different ways. We also determine an overall hotspot ranking for transport source regions into Australia, by integrating over the complete range of voyage risk weightings, and highlight changes in physical transport patterns over time (and associated changes in biosecurity risk). This framework provides an assessment of the strength of physical connectedness between regions. In an invasive species context, we envisage that the Australian case study will inform risk-based biosecurity processes as mandated in the Australian Government Biosecurity Act 2015, and assist with cost effective assignment of biosecurity effort.

## Methods

For physical international transport into Australia we obtained (1) shipping data from the Australian Government Department of Agriculture (formerly the Department of Agriculture, Fisheries and Forestry), and (2) flight information data from OAG Aviation (www.oag.com), over the time period 1999–2012. These data comprised a total of 142070 voyages by sea and 1042807 flights. Flights were separated into predominantly cargo flights (zero recorded passengers), and passenger flights.

Flight data were available at a level aggregated monthly by flight number, i.e., the dataset recorded, for each flight number visiting an Australian airport from overseas: each airport visited during the journey, the number of times that journey occurred within the given month, and the number of seats available for passengers on these flights. Some flight numbers consisted of only a single journey leg (e.g., Auckland to Melbourne), while others consisted of multiple stopovers (e.g., London to Sydney via Singapore). Specific dates or times on which these journeys occurred, or plane histories, were not available.

Shipping data included every vessel that travelled to Australia from overseas during the study period, and the port they visited most recently before travelling to Australia. Vessel history prior to the most recent port was not available. A detailed analysis of these shipping data, including types of vessels, ports visited, changes in traffic patterns over time, and associated ballast water discharge is available in [20].

Each port and airport listed in the flight and shipping datasets was assigned to a geopolitical region (state/province/territory within each country) within which it was physically located, using a global spatially referenced geopolitical database in ArcGIS. Some small countries, e.g., Singapore, did not have suitable sub-national units and thus were aggregated at the country level. Locations of ports and airports were sourced from Lloyds (www.lloydsmiu.com) and OAG Aviation (www.oag.com) respectively. The geopolitical database was adapted from the World Administrative Divisions layer package (ESRI 2012), edited to suit the ISO country naming conventions.

Traffic volumes into Australia from each geopolitical region over each year of the study period were aggregated. This included number of ships arriving, number of passenger and cargo planes arriving, and the number of passengers on those passenger flights.

### Weighted risk calculation

We constructed a weighted risk metric, so as to combine transport volumes of different voyage types (i.e., passenger flights, cargo flights, or ship voyages) into a single metric of the relative risk each donor region presents. We assume that, in a particular context, the risk presented by each voyage type may be different, e.g., a particular alien species may be more likely to be transported successfully by air than by sea, due to the difference in journey duration. We also assumed these relative risks were consistent across all flights or voyages, and not dependent on the source or destination location. Each voyage type is assigned a weighting: *w*_*p*_ for passenger flights, *w*_*c*_ for cargo flights and *w*_*s*_ for ship arrivals, and we can then use physical transport data to rank source regions based on their weighted cumulative impact. These individual weightings *w*_*p*_, *w*_*c*_ and *w*_*s*_ encode how much more or less likely one transport type is to be carrying a biosecurity threat than other transport types. For example, if, on average, we expect a ship to be three times as likely as a passenger flight to be carrying an alien species, then *w*_*s*_ would be three times *w*_*p*_. The weightings can in practice be any non-negative value, with the most important factor being their ratios. For ease of interpretation, in this paper we consider primarily cases with one weighing set to 1, so that the remaining weightings can be interpreted directly as the number of times more or less likely vessels of other types are to be carrying cargo of biosecurity concern (e.g., alien species). A weighting of zero would mean that you do not consider that pathway to contribute any transport risk in the context in question. The weighted cumulative impact from a given source region is *I*: = *w*_*p*_ × #passenger flights + *w*_*c*_ × #cargo flights + *w*_*s*_ × #ships.

We present two example scenarios under this framework: (1) with equally weighted voyages *w*_*p*_ = *w*_*c*_ = *w*_*s*_ = 1, and (2) with cargo voyages, particularly ships, weighted relatively highly (*w*_*s*_ > *w*_*c*_ > >*w*_*p*_). These two scenarios correspond to passenger flight, and cargo dominated risk, respectively.

To determine how variation in weightings resulted in variations in the highest risk regions, we determined the top 5 regions for each combination of weightings with *w*_*p*_ = 1 fixed, and *w*_*c*_ and *w*_*s*_ each between 0 and 50 (i.e., *w*_*c*_ and *w*_*s*_ values at each point of a square grid, with grid size 0.01). For each of these parameter combinations, we also determined the proportion of total weighted cumulative impact assigned to these top five source regions, to give an idea of how ‘top heavy’ the rankings are for a given parameter combination. Weightings for which the top 5 source regions remained consistent were displayed on a ‘phase-change plot’.

### Online tool

An online tool was developed using the ‘Shiny’ package [34] in the R statistical programming environment [35]. Users can choose to view data for transport into Australia in a given year, and choose weightings for each voyage type (*w*_*p*_, *w*_*c*_, *w*_*s*_). The resulting output is: (1) a map showing relative risk from each location, (2) a list of the regions providing the most risk, and (3) a plot indicating how these rankings have changed over time. In the online tool, flights can be assessed on last port of call, as with the examples in this manuscript, or proportionally over each previous stop (by flight number), e.g., a single-code flight travelling from London to Sydney via Singapore was either assigned as from Singapore (last port of call) or equally between London and Singapore. Ship voyages are only able to be assessed from their last port of call before travelling to Australia.

The tool can be viewed online at: https://robertcope.shinyapps.io/physical_network, and downloaded as R code from https://github.com/robert-cope/physical_network/.

### Overall hotspot ranking

To determine an overall risk ranking for source regions of traffic into Australia, we integrated rankings across a broad parameter space. We explored the range of parameter space from *w*_*p*_ = *w*_*c*_ = *w*_*s*_ = 1, to the smallest weighting for each parameter such that the ranking under that weighting (with the other weightings set to 1) was the same as if that was the only transport mode considered (i.e., in mathematical terms, the *infimum* of the set of weightings for which this is the case). For example, shipping weighting *w*_*s*_ ranged from 1, to the value of *w*_*s*_ for which the overall ranking (with *w*_*p*_ = *w*_*c*_ = 1) was the same as a ranking based entirely on shipping traffic. The non-uniform parameter ranges were chosen so that the overall ranking assessed the contributions of each vessel type equally; if the same range was used for each parameter then passenger flight traffic would dominate as there were many more passenger flights than other traffic types. The overall score assigned to a source region was *I*: = *w*_*p*_ × #passenger flights + *w*_*c*_ × #cargo flights + *w*_*s*_ × #ships (as defined earlier). We explored all unique ratios of parameter weightings within the parameter space: one weighting choice was set to be equal to 1, and then 10,000 uniformly chosen combinations of the other two weightings were used, and this was repeated for each choice of fixed weighting for a total of 30,000 points. For each source region, we recorded their aggregate ranking value, i.e., the highest risk region scores 1, the second highest risk region scores 2, etc., and thus the overall highest risk regions were those with minimal aggregate ranking value (a hypothetical source region that was highest risk at every point would have cumulative ranking value of 30,000). We also counted the proportion of parameter values for which a given source region was one of the top 1, 5, 10 or 50 highest risk source regions.

## Results

### Example: *w*_*p*_ = *w*_*c*_ = *w*_*s*_ = 1

**Fig 1:**
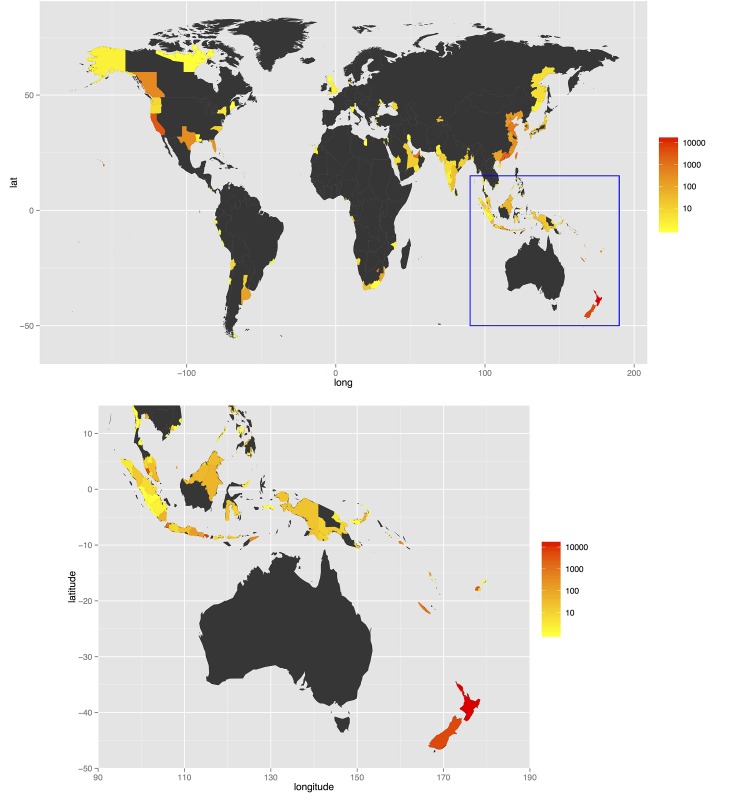
Combined air and sea traffic volume into Australia during 2012, as measured by weighted cumulative impact, *I*: = *w*_*p*_ × #passenger flights + *w*_*c*_ × #cargo flights + *w*_*s*_ × #ships. In this example each traffic type is weighted equally: *w*_*p*_ = 1, *w*_*c*_ = 1 and *w*_*s*_ = 1. Maps created in R 3.1.0 using geographic data curated in ArcGIS 10.2.

As a baseline scenario, we considered each voyage type to be weighted equally (i.e., *w*_*p*_ = *w*_*c*_ = *w*_*s*_ = 1), based on data from 2012. The highest contributing regions were dominated by those with greatest passenger flight traffic, in particular the North Island of New Zealand, Singapore, Bali (Indonesia), and Hong Kong (Table 1, Fig 1). Changes in overall ranking between 1999 and 2012 under these weightings were minor. Under this scenario, passenger flight traffic is overall the most dominant transport type, given that the numbers of passenger flights for high-traffic regions is much higher than for shipping and cargo flight traffic.

**Table 1:** Top 10 source regions of air and shipping traffic into Australia during 2012, based on weighted cumulative impact *I*: = *w*_*p*_ × #passenger flights + *w*_*c*_ × #cargo flights + *w*_*s*_ × #ships, with equal weightings on each traffic type: *w*_*p*_ = 1, *w*_*c*_ = 1 and *w*_*s*_ = 1. ‘1999 rank’ denotes the rank held by the source region when the same analysis was performed on 1999 traffic data, e.g., under this weighting Hong Kong had third highest *I* with these weightings in 1999.

Region	passenger flights	cargo flights	ships	weighted cumulative impact (*I*)	1999 rank
New Zealand—North Island	15546	426	537	16509	1
Singapore	10812	251	1410	12473	2
Indonesia—Bali	6600	0	42	6642	4
Hong Kong	5296	177	342	5815	3
New Zealand—South Island	4762	261	110	5133	5
Malaysia—Selangor	4317	127	105	4549	6
United States—California	3106	0	46	3152	7
Thailand—Samut Prakan	2821	17	3	2841	8
Fiji—Western	2608	0	5	2613	9
Papua New Guinea—Capital District	2193	183	157	2533	11

### Example: *w*_*p*_ = 1,*w*_*c*_ = 20,*w*_*s*_ = 50

**Fig 2:**
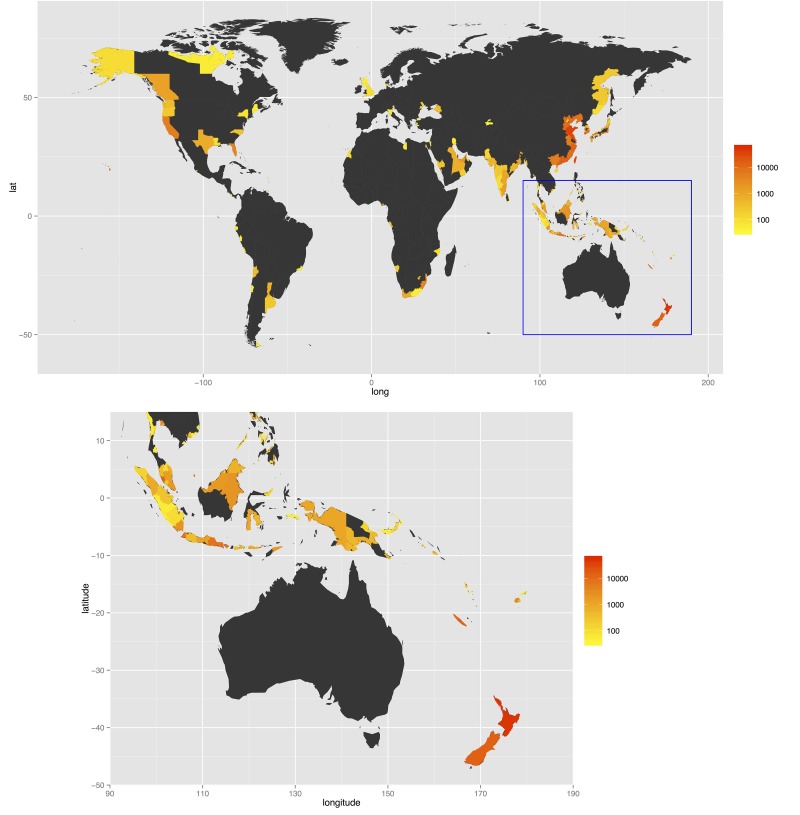
Combined air and sea traffic volume into Australia during 2012, as measured by weighted cumulative impact, *I*: = *w*_*p*_ × #passenger flights + *w*_*c*_ × #cargo flights + *w*_*s*_ × #ships. In this example cargo traffic is weighted heavily: *w*_*p*_ = 1, *w*_*c*_ = 50 and *w*_*s*_ = 20. Maps created in R 3.1.0 using geographic data curated in ArcGIS 10.2.

When cargo flights and vessels were weighted highly, with *w*_*p*_ = 1, *w*_*c*_ = 20 and *w*_*s*_ = 50, the relative rankings of regions differed significantly from the previous equal-weightings scenario. Regions with high levels of traffic for all three types, including Singapore and the North Island of New Zealand, remained highly ranked (relative to the *w*_*p*_ = *w*_*c*_ = *w*_*s*_ = 1 rankings), but regions with few flights and very many cargo ships gained higher ranks, such as Shandong (China) (Table 2, Fig 2). In some source regions, there had been a marked change in ranking over the period 1999–2012, with Shandong (China) moving from 70th to 3rd due to an increase in shipping traffic from 27 vessels in 1999 to 737 vessels in 2012, and similarly Jiangsu (China) from 36th to 5th (67 ship voyages in 1999 to 597 in 2012).

**Table 2:** Top 10 source regions of air and shipping traffic into Australia during 2012, based on weighted cumulative impact *I*: = *w*_*p*_ × #passenger flights + *w*_*c*_ × #cargo flights + *w*_*s*_ × #ships, with cargo traffic weighted heavily: *w*_*p*_ = 1, *w*_*c*_ = 20 and *w*_*s*_ = 50. ‘1999 rank’ denotes the rank held by the source region when the same analysis was performed on 1999 traffic data, e.g., under this weighting the Shandong region of China had 70th highest *I* under these weightings in 1999.

Region	passenger flights	cargo flights	ships	weighted cumulative impact (*I*)	1999 rank
Singapore_Singapore	10812	251	1410	86332	1
New Zealand_North Island	15546	426	537	50916	2
China_Shandong			737	36850	70
Taiwan (Province of China)	634		613	31284	3
China_Jiangsu			597	29850	36
Hong Kong	5296	177	342	25936	5
Korea (the Republic of)_Jeonranamdo			486	24300	16
China_Shanghai	1557	104	390	23137	26
China_Guangdong	1624		278	15524	18
New Zealand_South Island	4762	261	110	15482	6

### Phase changes & overall trend

**Fig 3:**
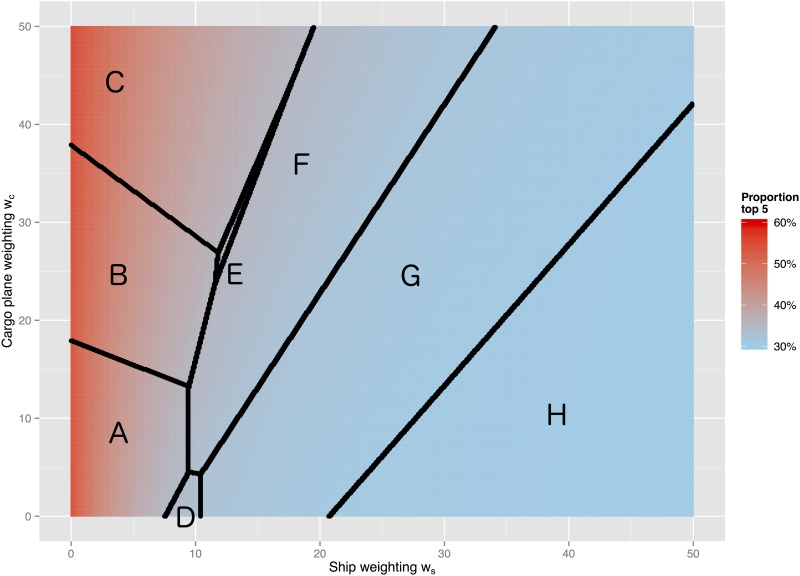
Phase-change diagram. *w*_*p*_ = 1 is fixed. Each line depicts the combination of weights that result in the composition of the top 5 highest risk source regions changing. Essentially, when moving upwards across a phase-change line, a source region with more cargo flights is entering the top 5, and when crossing a line towards the right, a source region with more shipping traffic is entering the top 5. Labels correspond to the source region groups in Table 3. Background colour shows, for a given parameter combination, the proportion of total weighted cumulative impact that originated from the top 5 source regions in 2012.

The threshold values at which the relative importance of locations switches was calculated for each pair of locations. The top 5 source regions were the source of c. 60% of all weighted cumulative impact when *w*_*s*_ was small, but this decreased to c. 30% as *w*_*s*_ increased (Fig 3).

**Table 3:** The top 5 source regions for physical connectedness into Australia within each section of weightings phase-space, with groups corresponding to the labels in Fig 3.

A	B
New Zealand North Island	New Zealand North Island
Singapore	Singapore
Indonesia—Bali	New Zealand South Island
Hong Kong	Hong Kong
New Zealand South Island	Malaysia—Selangor
C	D
New Zealand North Island	Singapore
Singapore	New Zealand North Island
New Zealand South Island	Hong Kong
Hong Kong	Indonesia—Bali
PNG National Capital District	China—Shandong
E	F
Singapore	Singapore
New Zealand North Island	New Zealand North Island
Hong Kong	Hong Kong
New Zealand South Island	New Zealand South Island
China—Shanghai	China—Shandong
G	H
Singapore	Singapore
New Zealand North Island	New Zealand North Island
China—Shandong	China—Shandong
Hong Kong	Taiwan
Taiwan	China—Jiangsu

For all combinations of weightings, the total weighted cumulative impact into Australia across all regions increased between 1999 and 2012. The greatest increases were when only passenger flights had non-zero weights (i.e., *w*_*p*_ = 1,*w*_*c*_ = *w*_*s*_ = 0), with an increase of 93% above 1999 levels. Greater proportional increases occurred with increasing ship weightings than with increasing cargo flight weightings. Although a few individual regions had extremely large increases in shipping traffic across the study period, these were counterbalanced by the majority of regions having much smaller changes e.g., with *w*_*p*_ = 1,*w*_*c*_ = 0,*w*_*s*_ = 50, the total weighted cumulative impact increased by 51% between 1999–2012.

### Overall hotspot ranking

We integrated over a range of parameter choices to determine an indication of overall source region importance. Parameter bounds were chosen to be the minimum value for a given weighting such that when the other weightings were set to 1, the overall ordering was dependent only on that parameter. The parameter bounds were *w*_*p*_ = 68, *w*_*c*_ = 1661, and *w*_*s*_ = 700. 30,000 parameter weightings were chosen uniformly such that each trio of parameter ratios was unique. The aggregate rankings of each region were recorded, along with the proportion of parameter space in which they were among the top 1, 5, 10, or 50 highest risk regions (Table 4). The highest risk source regions overall were the North Island of New Zealand and Singapore, sharing the top overall ranking almost equally across the parameter space. Source regions that had significant representation of all 3 traffic types ranked highly overall, including Hong Kong, the South Island of New Zealand, Shanghai (China), Selangor (Malaysia), and the Papua New Guinea Capital District. Source regions that had very high levels of one particular traffic type, such as Shandong (China) and Bali (Indonesia), with high levels of shipping and passenger flights, respectively, were also among the top overall risk hotspots.

**Table 4:** Top 20 source regions of air and shipping traffic into Australia in 2012, when rankings were determined by weighted cumulative impact (*I*: = *w*_*p*_ × #passenger flights + *w*_*c*_ × #cargo flights + *w*_*s*_ × #ships) integrated over possible values of *w*_*p*_, *w*_*c*_ and *w*_*s*_. The aggregate ranking is the sum of all ranking positions for that source region over 30,000 combinations of parameter values, and the proportions of parameter values for which the source region is part of the top 1, 5, 10, and 50 ports are also shown.

Region	Aggregate ranking	Top 1	Top 5	Top 10	Top 50	Passenger Flights	Cargo Flights	Ships
Singapore—Singapore	45310	49.47	100	100	100	10812	251	1410
New Zealand—North Island	46880	50.53	100	100	100	15546	426	537
Hong Kong	132603	0	81.23	100	100	5296	177	342
New Zealand—South Island	193855	0	65.92	86.74	100	4762	261	110
China—Shanghai	254062	0	0	88.44	100	1557	104	390
Papua New Guinea—National Capital District	290617	0	14.16	66.07	100	2193	183	157
Malaysia—Selangor	299939	0	10.39	62.45	100	4317	127	105
Taiwan (Province of China)	403927	0	29.43	52.27	100	634	0	613
China—Guangdong	434398	0	0	13.32	100	1624	0	278
Japan—Tiba	493813	0	0	0.27	100	1415	0	237
Republic of Korea—Incheon Gwang’yeogsi	602008	0	0	26.12	100	1105	105	62
New Caledonia	629114	0	0	0	100	587	0	229
Indonesia—Bali	635520	0	29.64	53.33	84.03	6600	0	42
China—Shandong	652440	0	41.49	53.99	73.77	0	0	737
China—Fujian	706297	0	0	8.75	95.59	0	105	136
China—Jiangsu	731585	0	17.60	46.71	71.89	0	0	597
Republic of Korea—Jeonranamdo	794900	0	0	31.20	70.53	0	0	486
United States—California	810192	0	0	21	72.67	3106	0	46
Indonesia—Banten	838953	0	0	0	100	881	0	111
United States—Hawaii	902239	0	0	7.12	81.75	933	104	3

## Discussion

Border security policy urgently requires common frameworks for interpreting and analyzing risk-based transport of individuals and commodities [36]. We have developed a framework for comparing physical connectedness between regions based on weighted aggregates of shipping and air traffic, and demonstrated this framework by using Australia as a case study.

The framework we have developed is novel for three key reasons. First, it combines traffic volumes across pathways to provide a single comprehensive measure describing the physical connectedness between geopolitical regions across multiple pathways. Secondly, it uses variable weightings for each transport pathway, so that the resulting output for a given weighting is easily customizable to suit a given situation, and the integrated ranking over all weightings fully encapsulates physical connectedness between regions without making strong assumptions on the relative contributions of each traffic type. Finally, it allows the assessment of changes in physical connectedness between geopolitical regions over time, which can assist with understanding changes in border security risk, and inform effective assignment of border security effort given these changing conditions.

Quantifying physical connectedness between regions provides useful data for identifying transport-risk hotspots when presented alone, but may also be effectively combined with other analyses. For example in an invasion context, if a particular species is of concern, there are a number of possible complementary approaches: (i) weightings could be chosen to suit the biology of the species (e.g., how long it can survive transport; the relevant transport commodities, etc.); or (ii) regions could be filtered pre- or post-hoc to include only those from which a species could be transported. The second approach, however, should be treated with caution, as it is possible that species may arrive via intermediate locations within which they may not naturally occur. Connectivity could also be combined with measures of environmental similarity to highlight risk that a transported species could survive in the new location. The flexibility of this approach means that it may be easily adapted to a range of situations.

The choice of transport mechanism weightings has a significant impact on relative risk between regions. A number of regions, including most prominently Shandong and Jiangsu (China), have significant amounts of shipping traffic but very little (or no) air traffic. These regions would present much higher risk for species capable of surviving the duration of a seaborne journey, particularly insects with egg or larval stages [37]. Conversely, there were a few regions, including Bali (Indonesia) and California (US), with significant amounts of air traffic but much less seaborne traffic. However, the regions that are likely to be of primary concern over the broadest range of potential alien species are those with high levels of traffic across all voyage types. This was demonstrated when rankings were integrated over the full range of weighting combinations, and the resulting highest risk regions tend to fit into clear patterns: the highest risk regions dominated by those with high levels of traffic of all types, including regions with both key international air hubs and heavy shipping traffic (Singapore, Hong Kong, Shanghai, Selangor), and regional trade partners (both Islands of New Zealand, PNG capital district). The remainder of the top 20 source regions (Table 4) consists either of very high levels of traffic along a single pathway (Passenger flights: Bali, California. Ships: Shandong, Jiangsu, Jeonranamdo), or relatively high traffic levels along two of the three pathways. We observed that different pathways have been subject to inconsistent increases in traffic volumes over time, with the greatest global increases in volume being in passenger flights. However, shipping traffic from a number of regions, particularly in China, has increased at a rate far greater than the overall trend. It is likely that invasion risk for species capable of being transported by sea from these regions has increased substantially, and as such biosecurity managers and policy makers should ensure that the distribution of biosecurity effort has changed in line with these changing physical transport patterns and associated risks.

By considering this physical network purely in terms of flights and shipping, we are abstracting away from the specific consideration of particular invasion pathways: intentional transport (including illicit smuggling), unintentional live stowaways, or introduction alongside a commodity. Each of these pathways is going to have different interactions with physical transport mechanisms, with factors such as voyage duration, environmental conditions throughout the voyage, and biosecurity controls each playing an important role. For example, unintentional stowaways or accidental introduction alongside a commodity are perhaps most likely to be associated with shipping traffic or cargo flights, given that all passenger flights into Australia have strict controls to prevent the transport of stowaways such as insects. Intentional transport, however, appears to be primarily mediated through air transport in Australia, likely due to the shorter duration of journeys, with many recorded incidences of wildlife smuggling detected by customs officers at Airports [38].

The two highest risk regions, Singapore and the North Island of New Zealand, provide contrasting examples of biosecurity risk. Singapore is a global hub for both air and sea traffic, being both the world’s busiest port by arrival tonnage [39] and being the 11th highest air passenger traffic country in 2013 (OAG) despite being only a single city with a relatively small population. In contrast, New Zealand is a regional trade partner with Australia, with similar agricultural and horticultural industries. A recent example of an alien species intercepted from New Zealand is the tomato-potato psyllid, intercepted twice between 2009–2012 alongside fruit, vegetable and nursery stock [40]. The psyllid is associated with Zebra chip disease, first confirmed in New Zealand in 2008 having previously been detected in the US. While both Singapore and New Zealand are the source of historically recorded alien species (e.g., [31]), intercepted as illegal imports or stowaways, they do not stand out as the most significant regions, and in particular New Zealand is the source of relatively few alien interceptions. And more generally it is important to note that future invasion patterns may not necessarily align with past invasion patterns. For example, many species established as invasive in Australia and globally were introduced by acclimatisation societies in the 19th and early 20th centuries [41], a pathway that no longer exists given changes in social attitudes. However, despite the end of this practice, vertebrate invasions are ongoing and indeed increasing globally [9, 41].

There are a number of reasons why these regions with high physical connectedness are not those that present the highest realised invasion risk. Singapore is geographically small and highly urbanized, as such it is highly unlikely that any alien species transported from Singapore are native. Cargo entering Australia from Singapore has likely undergone multiple stages of biosecurity processing prior to entry. Instead, one of the most pertinent biosecurity risks presented by Singapore is in the global spread of emerging infectious diseases via international air passenger traffic, due to the combination of strong connectedness between countries within south east Asia alongside a multitude of intercontinental connections [42]. In contrast, New Zealand is relatively geographically remote and is not a global transport hub. Given this, the greatest biosecurity risks presented by New Zealand are likely the transport of pests or diseases capable of disrupting agriculture. Any alien species arriving from New Zealand are likely to be established within New Zealand (alien or native) before subsequent transport to Australia. However, New Zealand has perhaps the most comprehensive biosecurity policy approach in the world [30], including an emphasis on pest-free export, and as such the transport of pests or diseases from New Zealand is much less likely than from countries with less stringent biosecurity controls. This may provide an opportunity for the use of pre-approval or measures directed towards cost-effective assignment of biosecurity effort.

The approach taken here rests on a number of key assumptions. First, we assumed that the relative risk of a flight or voyage transporting an alien species was independent of the source or destination region, or any other factors, and thus that transport risk relates only to the number of flights or voyages. It is conceivable, and indeed highly likely, that different source regions could provide differing baseline transport risk profiles, perhaps due to customs regulations imposed on vessels departing that region, or due to higher or lower availability of alien species to be transported (either native to the region in question, or due to transport/trade hubs). Further, the ability of a transported species to survive at its destination likely depends on environmental conditions, i.e., if conditions are similar between the source and destination. As such, this framework quantifying physical connectedness between regions should be analysed carefully alongside additional models and data relevant to the biosecurity context of interest.

Secondly, ships and flights were generally recorded as originating from their last port of call. In practice, planes or ships themselves, or the transported passengers or cargo, can commence their journey at a previous port of call, potentially taking on alien species at each port visited. In some cases, particularly for air traffic, flights with multiple stops before arriving into Australia are all covered by a single flight number and as such users of the online tool may consider these flights as spread over their whole path rather than restricted to the last port of call. But these data do not cover cases where passengers may have travelled via a host country on flights that do not share a flight number, or for the initial source of cargo or ships that arrived via an intermediate destination. When long-term data are available detailing the travel histories of ships or cargo (e.g., [22]), it might be possible for a future study to investigate in detail the potential origins of the transport of alien species. However, in this study we restrict our analysis to direct physical connectedness between regions, a more flexible approach relevant in a range of biosecurity contexts.

Air traffic and sea traffic have many different characteristics that may contribute to transport risk. One of the clearest differences is in travel time: a journey, e.g., Singapore to Melbourne, that takes less than 8 hours by air may take c. 15 days by sea. A species capable of surviving 8 hours on a plane but not 15 days on a ship, for example, would thus be more likely to be introduced by air. This should contribute to the choice of weightings assigned to each traffic type. However, this does not mean that air traffic is always going to be the highest risk pathway, as other species may be able to survive a seaborne journey, or be transported alongside commodities that travel by ship. Weightings should be chosen carefully, taking into account any factors relevant to the situation.

Finally, since Australia is an island nation, international physical transport network pathways are limited to air and sea based transport. If an analysis along these lines was to be considered elsewhere, it may be necessary to consider land-based dispersal as an additional potential pathway [7].

The introduction and establishment of alien species presents significant economic and environmental costs worldwide. In this study, we have described a framework to quantify the physical connectedness between geopolitical regions, with Australia as a case study, based on weightings that can be chosen to suit pathways of interest. We have highlighted the source regions with the highest physical connectedness to Australia: the North Island of New Zealand, and Singapore. These data provide a baseline model for the transport of alien species, and more broadly of transport of individuals and commodities into Australia and associated biosecurity risks. We envisage that this framework should be a valuable tool for biosecurity policymakers, and provides an example of a approach that could be applied for other networks worldwide.
